# Effect of different cold application materials on pain during chest tube removal: three-arm randomized controlled clinical trial

**DOI:** 10.4314/ahs.v21i3.38

**Published:** 2021-09

**Authors:** Dilara Soydan, Gülay Altun Uğraş

**Affiliations:** Mersin University, Turkey

**Keywords:** Chest tube removal, cold application, pain, nurse

## Abstract

**Background:**

Chest tube causes severe pain during removal because it attaches to the endothelium in the chest cavity.

**Objectives:**

This study aimed to determine the effectiveness of cold application with ice pack and gel pad in the control of pain experienced during chest tube removal.

**Methods:**

The sample of prospective, parallel three-arm (1:1:1), randomized controlled clinical trial consisted of 180 patients in two experimental groups (ice pack/gel pad) and one control group. The primary outcome was effect of cold application materials on severity of pain during chest removal. Secondary outcomes were duration of cold application and analgesic requirements of the patients.

**Results:**

The study found that the cold application using either of the materials reduced the severity of pain and the need for analgesics after the removal of chest tube compared to the control group (p<0.05). But cold application with ice pack allowed the skin to drop to the temperature effective in pain control in a shorter time than gel pad application (p<0.05).

**Conclusions:**

Despite entirely covering the area around the chest tube, the gel pad was more disadvantageous than ice pack in pain control due to the longer duration of cold application.

## Introduction

Chest tube used in the treatment of acute or chronic problems caused by thoracic surgery or trauma allows the air and fluid to be drained from the pleural space.^1^ Although chest tube application is an important therapeutic intervention, it causes severe pain that reduces the comfort and satisfaction of the patient during removal.^2^,^3^ During the removal of chest tube, the individual experiences pain caused by adherence to the tissue which the tube contacts with and separation of adhesions due to withdrawal.^1^,^3^–^5^ Patients describe chest tube removal as a painful procedure.^2^,^3^,^5^–^7^ Analgesic agents administered during chest tube removal are frequently used in the treatment of acute pain.^2^,^8^,^9^ However, severe pain management requires the use of non-pharmacological agents in addition to pharmacological agents because the patient's response to pharmacological treatment is varied and there is often no complete control of pain with these agents.^10^ One of the non-pharmacological methods used in pain management is cold application. Cold application creates an analgesic effect by reducing the oxygen and nutrient requirement of tissues by slowing the metabolism; eliminating pressure and tension on nerve endings by limiting inflammation, spasm and edema; and slowing or blocking the transmission rate of peripheral nerves. In addition to this, it stimulates the touch receptors with the door-control theory, increases the release of endogenous opioids and thus decreases the pain.^3^–^6^,^11^–^13^ Research has shown that cold application is effective in controlling pain occurring during chest tube removal process and that less analgesic is needed due to cold application in this process. There are study reports on cold application with ice pack or gel pad^2^–^4^, but there are no currently available, previous studies results on the comparative effect of two different cold application materials on pain.

The material used must be in completely contact with the skin so that cold application can be effective in pain control. In cold application with ice pack commonly used in clinics, it is possible that the material does not come into completely contact with some areas of the skin around the chest tube and this may lead to less effective cold application in pain control. On the other hand, in cold application with gel pad, which completely surrounds the chest tube and conforms to the body curves, whether the skin temperature required for the cold application to be effective is reached or not has not been tested so far.^2^,^5^–^7^ This study was aimed to determine which cold application material is most effective in pain control of patients during chest tube removal process. The following hypotheses are proposed:

H1 hypothesis: There is difference between two cold application materials affects the severity of pain during chest tube removal.

H2 hypothesis: There is difference between two cold application materials duration of cold application during chest tube removal.

H3 hypothesis: There is difference between two cold application materials analgesic requirements of the patients during chest tube removal.

## Methods

### Study design

This study was a prospective, parallel three-arm [1:1:1], randomized controlled clinical trial at the Thoracic Surgery Unit of a university hospital in Turkey between September 2016 and November 2017.

This trial was registered with ClinicalTrials.gov (NCT04200859) and approved by the Mersin University Clinical Researches Ethics Committee (Number/date: 219/2016). Written and verbal consent was obtained from all the patients, too.

### Participants

**Figure 1:**
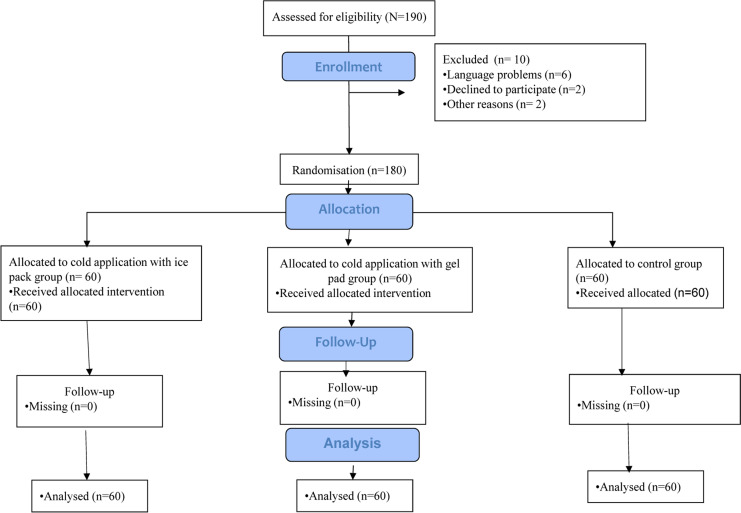
CONSORT flow diagram for this trial

The study sample consisted of 180 patients aged ≥18 years who underwent chest tube at the Thoracic Surgery Unit. Patients included in the study were conscious, orientated and cooperated, who could speak and understand Turkish, had no psychiatric disease, had chest tube and had stable general status, aged 18 and over, who had written and oral permission to participate in the study (n=180). A total of 10 patients were excluded from the sample because six of them had language problems, two of them did not agree to participate in the study and it was impossible to communicate with two of the patients due to their psychiatric problems (Figure 1).

### Sample size

In order to calculate the sample size, a pain reduction of at least 0.5 standard deviation in the Numerical Rating Scale (NRS) by cold application with gel pack or ice pack was taken as statistically significant, and Demir and Khorshid's study was taken as the reference for this study with 5% Type I Error and 80% test power.^2^ The sample size was finally calculated as 180 patients, with 60 patients in each group (experimental groups: ice pack group= 60 patients; gel pad group= 60 patients and one control group=60 patients).

### Randomization and allocation

The patients were invited to participate in the study between September 2016 and November 2017. Consenting eligible patients were randomly assigned to experimental groups and control group, according arrival sequence in blocks of 3 in a 1:1:1 ratio using the block randomization method. The randomization sequence was developed using a computer generated table of random numbers^14^ by a biostatistician who was not associated with the study. The researcher (D.S.) who was involved in the running of the study were not blinded. However, the biostatistician and researcher who interpreted the findings (G.A.U.) was blinded to the group allocation.

### Interventions

The study protocol was reviewed and approved by on Clinical Trials.gov (NCT04200859).

Conventional care: Routine analgesic drugs are not administered to patients before removal of the chest tube in thoracic surgery clinic. However, analgesic is performed according to the severity of pain after the procedure.

**Figure 2:**
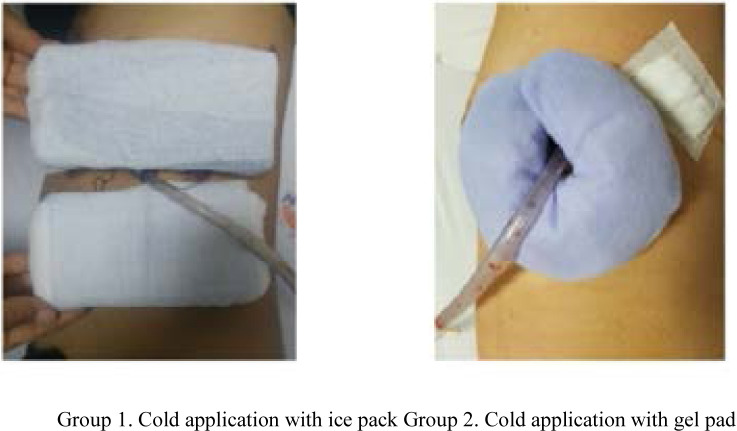
Use of cold application materials in the study Group 1. Cold application with ice pack Group 2. Cold application with gel pad

Cold application with ice pack/gel pad group: Before the chest tube was removed, two ice packs with a size of 15.5x9cm were inserted around the chest tube so as to make as much contact as possible. Before the chest tube was removed, a gel pad with a radius of 15 cm was completely inserted around the chest tube (Figure 2).

The temperature of the cold application materials (-10 °C) was measured with the barbecue thermometer (Barbecue Thermometer TB101, Mileegirl, China) before starting the intervention.

The primary outcome of this study was the effect of cold application materials on severity of pain.

Numerical Rating Scale: The scale was used to determine the severity of pain during chest tube removal process. Black and Matassarin-Jacobs developed the NRS^15^, and Tulunay and Tulunay tested its validity and reliability for use in Turkish.^16^ On this scale, patients are asked to describe their pain with numbers, with “0” indicating “no pain” and “10” indicating “the worst, unbearable pain”.^15^,^16^ Te severity of pain was measured using a NRS in this study since Yazıcı Sayın and Akyolcu reported that Turkish patients in the early postoperative period prefer NRS as it consists of numbers, and it is simple and easy to understand.^17^

The secondary outcomes of this study were duration of cold application and analgesic requirements of the patients.

The Patients Information Form was used to determine patients characteristics, duration of cold application and analgesic requirements of the patients. The form consisted of items designed to collect data about the patients such as age, gender, education status, cause of chest tube insertion, duration of cold application, presence of analgesic administration before and after chest tube removal, type of analgesic administered, time passed between the last analgesic time and chest tube removal time and time passed between chest tube removal time and the first analgesic administration time.

### Procedure

Before removal of chest tube, nurse researcher (D.S.) who is working in the thoracic surgery clinic as a nurse, explained aim of study and take informed consent all patients. Data were collected with Patient Information Form and NRS.

Patient Information Form was filled out for each using the information obtained from patients/patients' relatives, patient files and health professionals of the clinic and the patients' pain severity was measured with NRS.

The patients were randomly assigned by a computer program to either intervention groups (ice pack or gel pad) or control group. Before the chest tube was removed, nurse researcher (D.S.) made cold application with ice pack the first experimental group and cold application with gel pad the second experimental group. The control group did not receive any intervention. Since the cold application temperature should be reduced to 13.6°C in order to have local analgesic effect in both of the experimental groups^2^,^3^,^11^,^18^,^19^ the patient's skin temperature was measured at one-minute intervals by using an infrared non-contact thermometer (Microlife Non-Contact, Switzerland) with a wide measurement range (0–100°C) and a measurement time of three seconds. This application was terminated when the temperature was 13.6 °C and the chest tube was removed by the physician.

Pain severity of all the patients was measured with NRS immediately after and 15 minutes after chest tube removal.

### Data analysis

Data were analyzed on a computer.^14^ Descriptive statistics were presented using frequency, percentage, mean and standard deviation. Chi-square test was used to compare categorical variables. Independent t-test was used to compare two independent groups. One-way analysis of variance (ANOVA) was used to compare the means of more than two independent groups. Analysis of variance was used for repeated measurements. Finally, as further analysis, Tukey's test was used for multiple comparison analysis (post hoc) between groups. In data analysis of this study, the statistical significance level was set at p<0.05.

## Results

Table 1 shows the descriptive and clinical characteristics of the patients. It was found that the most common cause of a chest tube insertion in patients in all the groups was hemothorax (in 56.7% of the cold application group with ice pack, in 60% of cold application group with gel pad, and in 56.7% of the control group). There were no statistically significant differences between the groups in terms of age, gender, educational status, and chest tube insertion (p>0.05) (Table 1).

**Table 1:** Comparison of the descriptive and clinical characteristics of the patients (n:180)

Characteristics	Cold application with ice pack group	Cold application with gel pad group	Control group	Test	p
**Age, y, mean±SD**	49.4±16.0	53.7±16.4	47.6±18.5	2.05*	0.13

Gender, **n (%)**					

Female	21 (35.0)	22 (36.7)	18 (30.0)	0.65Ψ	0.72
Male	39 (65.0)	38 (63.3)	42 (70.0)		

**Level of Education, n (%)**					

Elementary Education	39 (65.0)	42 (70.0)	38 (63.3)	2.63Ψ	0.62
Secondary Education	9 (15.0)	10 (16.7)	14 (23.4)		
Higher Education	12 (20.0)	8 (13.3)	8 (13.3)		

**Cause of chest tube insertion, n (%)**					

Hemothorax	34 (56.7)	36 (60.0)	34 (56.7)		
Pneumothorax	10 (16.7)	12 (20.0)	15 (25.0)		
Pleural effusion	4 (6.7)	2 (3.3)	4 (6.7)		
Hydatid cyst	5 (8.3)	1 (1.7)	1 (1.7)		
Hemothorax and Pneumothorax	3 (5.0)	2 (3.3)	1 (1.7)	8.35Ψ	0.60
Other¥	4 (6.6)	7(11.7)	5 (8.2)		

¥ Other: pleural thickening, diaphragmatic hernia, diaphragmatic rupture, hyperhidrosis, thymoma, empyema.

* One-way ANOVA

Ψ Pearson's Chi-square test

### Comparisons of severity of pain

In this study, there were significant differences between the groups in terms of the NRS mean scores measured before, immediately after and 15 minutes after chest tube removal (p<0.05) (Table 2).

**Table 2:** The effect of cold application materials on pain severity

Time of pain severity measurement	Cold application with ice pack group^1^	Cold application with gel pad group^2^	Control group^3^	Test	p	Tukey's post-test
	mean±SD	mean±SD	mean±SD			
Before chest tube is removal^a^	6.3±2.2	7.5±2.0	6.1±2.2	7.29*	**0.001**	**2>1,3**
At chest tube removal ^b^	5.0±2.6	6.4±3.4	7.1±2.4	8.29*	**<0.001**	**3>1 2>1**
15 minutes after chest tube removal^c^	1.9±1.6	4.8±2.6	6.6±2.1	71.02*	**<0.001**	**3>2>1**
Test	63.80Ψ	14.30Ψ	3.40 Ψ			
**p**	**<0.001**	**<0.001**	**0.03**			
**Tukey's post-test**	**a>b>c**	**a>b>c**	**b>c >a**			

* One-way ANOVA

Ψ Repeated ANOVA

### In comparison between groups

The severity of pain prior to chest tube removal was significantly higher in patients treated with cold application with gel pad than the patients with treated with cold application with ice pack and than the patients in the control group (p<0.05). The severity of pain at the time of chest tube removal was significantly lower in the patients treated with cold application with ice pack than the patients with treated with cold application with gel pad and than the patients in the control group(p<0.05). Also, the severity of pain 15 minutes after chest tube removal was significantly lower in the patients treated with cold application with ice pack than the patients treated with cold application with gel pack (p<0.05). Finally, the severity of pain 15 minutes after chest tube removal was significantly lower in the patients treated with cold application with gel pad than the patients in the control group (p<0.05) (Table 2).

### In the in-group comparison

The severity of pain before chest tube removal in the patients treated with cold application with ice pack or gel pad was significantly higher than their pain severity scores measured immediately after and 15 minutes after chest tube removal (p<0.05). Similarly, the severity of pain in these patients at the time of removal was significantly higher than the pain severity scores measured 15 minutes after chest tube removal (p<0.05). In addition, the severity of pain at the time of chest tube removal in the control group patients was significantly higher than their pain severity measured before and 15 minutes after chest tube removal (p<0.05). Similarly, the severity of pain in these patients 15 minutes after chest tube removal was significantly higher than the pain severity measured before chest tube removal (p<0.05) (Table 2).

### Comparisons of duration of cold application and analgesic requirements

In this study, an average of 30 minutes of cold application was given to the patients with gel pad while an average of 18.9±2.6 minutes of cold application was given to the patients with ice pack. The application duration for the patients who received cold application with gel pad was significantly longer than the duration for the patients who received cold application with ice pack (p<0.05) (Table 3).

**Table 3:** The effect of cold application materials on duration of cold application and analgesic requirements of the patients

Time passed between the last analgesic time and chest tube removal time¥	Cold application with ice pack group^1^ mean±SD/ n (%)	Cold application with gel pad group^2^ mean±SD/ n (%)	Control group^3^ mean±SD/ n (%)	Test	p	Tukey's post-test
**Duration of** **cold** **application** (min-max)	18.9±2.6 (min:15-max:25)	30.0±0.0 (min=30-max=30)	-	-32.78*	**<0.001**	**2>1**

**Analgesic administration before chest tube removal**						

**Administered** **in 1–3 hours**	17 (54.8)	22 (73.3)	14 (46.7)	4.61Ψ	0.10	
**Administered** **in 4–6 hours**	14 (45.2)	8 (26.7)	16 (53.3)			
**Analgesic administration after chest tube removal**						
**Administered**	21 (35.0)	40 (66.7)	55 (91.7)	42.24 Ψ	**<0.001**	**1<2<3**
**Not** **administered**	39 (65.0)	20 (33.3)	5.0 (8.3)			
**Time passed between chest tube removal time and the first analgesic administration time** ¥						
**Administered** **in 1–3 hours**	10 (47.6)	31 (77.5)	48 (87.3)	13.40 Ψ	**0.001**	**1<2** **1<3**
**Administered** **in 4–6 hours**	11 (52.4)	9 (22.5)	7 (12.7)			

¥ Calculated based on the number of patients treated with analgesics only.

* Independent-sample t test

Ψ Pearson's Chi-square test

In addition, there was no statistically significant difference between the groups in terms of the status of analgesic administration before chest tube removal and the time elapsed between the last analgesic administration and chest tube removal (p>0.05). Prior to chest tube removal, the control group patients were given significantly higher doses of paracetamol but lower doses of diclofenac sodium and tramadol HCl than the patients treated with gel pad (p<0.05). Analgesic administration in the patients treated with cold application with ice pack after chest tube removal was significantly less than the patients treated with cold application with gel pad and the control group (p<0.05). Also, analgesic administration in the patients treated with cold application with gel pad after chest tube removal was significantly less than the control group (p<0.05). Analgesic administration within 1 to 3 hours after chest tube removal in patients treated with cold application with ice pack was significantly less than the patients treated with cold application with gel pad (p<0.05). Similarly, analgesic administration within 4 to 6 hours after chest tube removal in patients treated with cold application with ice pack was significantly less than the control group (p<0.05) (Table 3).

## Discussion

### Effect of cold application materials on severity of pain

In this study was determined that cold application with two different materials decreased the severity of pain after chest tube removal in comparison with the control group. In similar studies, the severity of pain after the procedure of patients who received cold application before chest tube removal was measured with Visual Analog Scale/NRS at different times, such as 5 and 15 minutes after the procedure, and it was shown that cold application was effective in pain control and reduced pain over time.^2^–^4^,^11^ Another study, however, found that cold application was not statistically effective in pain due to chest tube removal while it reduced clinical pain.^5^ The fact that cold application duration was limited to 15 minutes and skin temperature was not measured in the aforementioned study might have caused their cold application to be ineffective, which is a different result from this study.

An important result of this study is that ice pack was found to be more effective in reducing severity of pain compared to gel pad. This result may have been caused by the fact that the patients who received cold application with gel pad had higher pain before the procedure and the application was terminated in some patients without reaching the skin temperature (13.6 °C) required for cold application to be effective.

### Duration of cold application and analgesic requirements of the patients

In the present study, it was determined that cold application with ice pack reduced the temperature of the skin, which is effective in pain control, in a shorter time than the application with gel pad.

In order for cold application to show local analgesic effect, the skin temperature should drop below 13.6°C.^3^,^4^,^11^,^18^,^19^ While some studies limited the duration of cold application based on the skin temperature falling below 13.6°C^3^,^4^,^11^, like this study, cold application was performed for 15–20 minutes in some studies without a skin temperature measurement.^2^,^6^,^7^ However, unlike present study, previous studies did not compare the effect of two different cold application methods. In this study, cold application was terminated in 30 minutes in order to eliminate the possibility of a “rebound phenomenon”^18^ like replacement of vasoconstriction in the vessels caused by cold application in patients treated with gel pad by vasodilatation due to excessive drop in skin temperature. This result showed that ice pack was more effective than gel pad in reaching the effect of cold application in pain control in a short time.

In this study, the group who received the most analgesics after chest tube removal was the control group, followed by the experimental group treated with gel pad and the experimental group treated with ice pack, respectively. This result of the study showed that, no matter either ice pack or gel pad was used in pain control, cold application was more effective than no intervention in decreasing the need for analgesia and relieving pain.^2^,^6^ Although the effectiveness of ice pack^4^,^5^ and gel pad^2^,^3^,^6^,^11^ was previously demonstrated in studies on the effect of cold application in relieving pain, there are currently no reports on the comparison of two different cold application materials. An important result of this study is that ice pack was found to be more effective than gel pads in reducing the use of analgesics.

## Limitations

The limitations of this study; in some patients who had cold application with gel pad, cold application was terminated due to rebound effect in 30 minutes without skin temperature falling to 13.6°C.

## Conclusion

The results of this study showed that cold application with both materials decreased the pain severity and the need for analgesia after chest tube removal compared to the control group, but cold application with ice pack allowed the skin to drop to the temperature effective in pain control in a shorter time than gel pad application.

In interventions that cause acute pain, such as chest tube removal, non-pharmacological methods such as cold application as a part of independent functions of the nurse in addition to pharmacological methods should also be used in pain control. In pain management due to chest tube removal, nurses should develop a cold application protocol taking into account more effective cooling materials. Thus, analgesic requirements that may lead to serious respiratory complications such as respiratory depression in the patient may be reduced.

A further result of this study is that although gel pad was completely wrapped around the chest tube, it was more disadvantageous in pain control than ice pack probably due to the longer duration of cold application.
